# Supplementary material for: Characterization of Triterpene Saponin Glycyrrhizin Transport by Glycyrrhiza glabra
Source: Plants (Basel). 2022 May 5;11(9):1250. doi: 10.3390/plants11091250 (PMC9102456; doi:10.3390/plants11091250)
Supplement: Supplementary file 1 [file plants-11-01250-s001.zip › plants-1723621-supplementary.pdf]

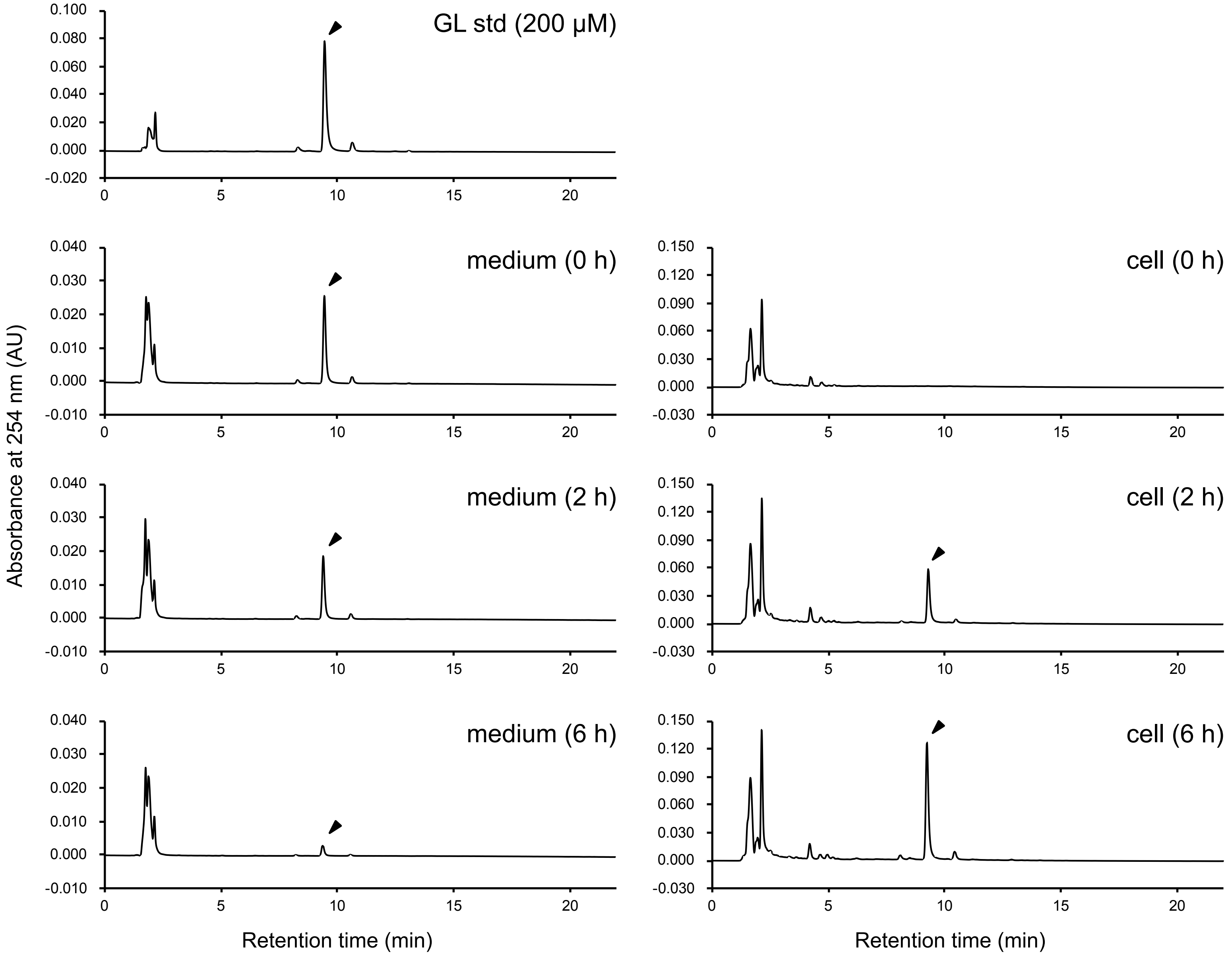

Figure S1. HPLC chromatogram of GL in the cultured medium and cultured cells of *G. glabra*.
GL was added to the cultured cells at a concentration of 100 µM. The culture medium and cell extracts were subjected to HPLC analysis.
